# Lipidome is a valuable tool for the severity prediction of coronavirus disease 2019

**DOI:** 10.3389/fimmu.2024.1337208

**Published:** 2024-05-10

**Authors:** Shan-Shan Zhang, Zhiling Zhao, Wan-Xue Zhang, Rui Wu, Fei Li, Han Yang, Qiang Zhang, Ting-Ting Wei, Jingjing Xi, Yiguo Zhou, Tiehua Wang, Juan Du, Ninghua Huang, Qinggang Ge, Qing-Bin Lu

**Affiliations:** ^1^ Department of Laboratorial Science and Technology and Vaccine Research Center, School of Public Health, Peking University, Beijing, China; ^2^ Center for Infectious Disease and Policy Research and Global Health and Infectious Diseases Group, Peking University, Beijing, China; ^3^ Department of Intensive Care Medicine, Peking University Third Hospital, Beijing, China; ^4^ Department of Epidemiology and Biostatistics, School of Public Health, Peking University, Beijing, China; ^5^ Pulmonary and Critical Care Medicine, Peking University Third Hospital, Beijing, China; ^6^ Department of General Surgery, Peking University Third Hospital, Beijing, China; ^7^ Department of Health Policy and Management, School of Public Health, Peking University, Beijing, China; ^8^ Key Laboratory of Epidemiology of Major Diseases (Peking University), Ministry of Education, Beijing, China

**Keywords:** COVID-19, SARS-CoV-2, lipid, LPC, immune, cytokine

## Abstract

**Objective:**

To describe the lipid metabolic profile of different patients with coronavirus disease 2019 (COVID-19) and contribute new evidence on the progression and severity prediction of COVID-19.

**Methods:**

This case–control study was conducted in Peking University Third Hospital, China. The laboratory-confirmed COVID-19 patients aged ≥18 years old and diagnosed as pneumonia from December 2022 to January 2023 were included. Serum lipids were detected. The discrimination ability was calculated with the area under the curve (AUC). A random forest (RF) model was conducted to determine the significance of different lipids.

**Results:**

Totally, 44 COVID-19 patients were enrolled with 16 mild and 28 severe patients. The top 5 super classes were triacylglycerols (TAG, 55.9%), phosphatidylethanolamines (PE, 10.9%), phosphatidylcholines (PC, 6.8%), diacylglycerols (DAG, 5.9%) and free fatty acids (FFA, 3.6%) among the 778 detected lipids from the serum of COVID-19 patients. Certain lipids, especially lysophosphatidylcholines (LPCs), turned to have significant correlations with certain immune/cytokine indexes. Reduced level of LPC 20:0 was observed in severe patients particularly in acute stage. The AUC of LPC 20:0 reached 0.940 in discriminating mild and severe patients and 0.807 in discriminating acute and recovery stages in the severe patients. The results of RF models also suggested the significance of LPCs in predicting the severity and progression of COVID-19.

**Conclusion:**

Lipids probably have the potential to differentiate and forecast the severity, progression, and clinical outcomes of COVID-19 patients, with implications for immune/inflammatory responses. LPC 20:0 might be a potential target in predicting the progression and outcome and the treatment of COVID-19.

## Introduction

Coronavirus disease 2019 (COVID−19) is an emerging infectious disease caused by the infection of severe acute respiratory syndrome coronavirus 2 (SARS-CoV-2). Most individuals infected with SARS-CoV-2 exhibit mild or moderate respiratory symptoms and undergo a self-limiting recovery process without necessitating specialized medical intervention (1). However, approximately 20% of the patients may develop to severe illness state and even die from the infection, especially in the elderly that warrant intensive medical care (2, 3). Numerous studies have explored the pathogenetic mechanism of SARS-CoV-2 infection, although it has been not yet fully elucidated (4). Given the various adverse outcomes of COVID-19, there is a need to conduct more research on the pathogenic mechanism and develop predictive tools for severe or fatal cases and targeted therapies.

Lipids have been acknowledged to be associated with viral infection in many regards (5–8). On the one hand, lipids are involved in various biological processes as the structural foundations of the membrane’s cells and viruses (9). They function as crucial elements in cell membranes and molecules for storing energy, playing a role in signaling processes (10). The host lipid synthesis pathway contributes a lot to the regulation and control of viral replication (11). Lipids serve as direct receptors or assist in the entry of various viruses on the cell surface or within endosomes (12). They also contribute to the formation and function of viral replication complexes and in the production of energy necessary for efficient replication of viruses (11). Moreover, lipids help in regulating the appropriate allocation of viral proteins inside the cell and play a role in the assembly, transportation, and release of viral particles (13, 14). On the other hand, viral infection disrupts host metabolism, depriving cellular energy and materials that support the viral infection phase, resulting in host metabolic dysregulation, including profound changes in lipid metabolism (15, 16). Viruses can cause a notable alteration in the lipid composition of host cells, impacting crucial energy pathways and facilitating in the viral infection process by utilizing the host cell’s metabolic resources to assist in different stages of viral replication and spread (11).

Recent research indicated that severe cases of COVID-19 were characterized by a systemic disruption in metabolic processes and widespread alterations in lipid composition. Severe COVID-19 patients were observed to exhibit elevated levels of plasma triglycerides (TAGs) and reduced levels of cholesterol esters (CEs) in comparison to mild COVID-19 patients (17). Moreover, Trovato et al. reported that lipid metabolism, particularly phosphatidylcholines and lysophosphatidylcolines, seemed strictly connected to immune response in COVID-19 (18). However, the role of lipids in COVID-19 remained unclear with a lack of emphasis on the importance of lipids in the immune response of COVID-19 patients.

Thus, this study aimed to depict the lipid metabolic profile of different COVID-19 patients and identify lipid biomarkers associated with the immune/cytokine responses of COVID-19 patients to provide new evidence on the progression of SARS-CoV-2 infection and aid in predicting the severity or prognosis of COVID-19 patients.

## Materials and methods

### Study design and population

This case–control study was conducted at Peking University Third Hospital. We enrolled laboratory-confirmed COVID-19 patients aged ≥18 years old whose imaging showed characteristic manifestations of COVID-19 pneumonia from December 2022 to January 2023. Exclusion criteria encompassed patients with lung tumor, bronchiectasis, interstitial lung disease, tuberculosis, pulmonary embolism, hepatitis, hyperthyroidism, and hyperuricemia, and those with a history of lung surgery were excluded. The severity of pneumonia was diagnosed according to the Diagnosis and Treatment of Novel Coronavirus Pneumonia (Version 9) released by the National Health Commission (19). The study protocol was approved by the Peking University Third Hospital Medical Science Research Ethics Committee (2023–014–02), and the informed consents were obtained from all patients.

### Data and sample collection

Demographic and clinical information were extracted from the medical records by a group of trained research assistants. The laboratory confirmation of COVID-19 adhered to the guidelines outlined in the Diagnosis and Treatment of Novel Coronavirus Pneumonia (Version 9) (19). The blood samples of laboratory-confirmed patients with COVID-19 were collected on the day of hospital admission and during the recovery phase of the surviving severe patients throughout their hospital stay.

### Metabolites extraction

A 10-μL sample was mixed with 190 μL of water, followed by the addition of 480 μL of extract solution (MTBE : MeOH = 5:1) containing an internal standard. After vortexing for 60 s, the samples were sonicated in an ice-water bath for 10 min and then centrifuged at 821 g for 15 min at 4°C. Next, 250 μL of the supernatant was transferred to a new tube. The remaining sample was mixed with 250 μL MTBE, followed by vortex, sonication, and centrifugation. Another 250 μL of supernatant was collected. This process was repeated once, and the supernatants were combined and dried in a vacuum concentrator at 37°C. The dried samples were then reconstituted in 100 μL of resuspension buffer (DCM : MeOH:H_2_O = 60:30:4.5), vortexed for 30 s, and sonicated for 10 min in an ice-water bath. The reconstituted samples were centrifuged at 13,800 *g* for 15 min at 4°C, and 35 μL of the resulting supernatant was transferred to a fresh glass vial for LC/MS analysis. A quality control sample was prepared by mixing an equal aliquot of the supernatants from all the individual samples.

### LC-MS/MS analysis

The UHPLC separation was carried out using a SCIEX ExionLC series UHPLC System. The mobile phase A consisted of 40% water, 60% acetonitrile, and 10 mmol/L ammonium formate. The mobile phase B consisted of 10% acetonitrile and 90% isopropanol, and 10mmol/L ammonium formate. The column temperature was maintained at 45°C, and the auto-sampler temperature was set at 6°C, with an injection volume of 2 μL. The AB Sciex QTrap 6500+ mass spectrometer was used for assay development, with typical ion source parameters including IonSpray Voltage at 5,500/−4,500 V, curtain Gas at 40 psi, temperature at 350°C, ion source gas 1 at 50 psi, ion source gas 2 at 50 psi, and DP at ±80V. The measurement of the specific compounds was carried out using Biobud-v2.1.4.1. The exact quantity of each lipid was determined by analyzing the peak area and the actual concentration of the internal standard from the same lipid class.

### Laboratory test for immune/cytokine indexes

The detection of lymphocyte and its subsets in patients’ peripheral venous blood samples was conducted using fluorescent monoclonal antibody kits (Beijing Tongshengshidai Biotechnology Co., Ltd, Beijing, China; Cat. no. 20153402073). The cytokine in serum samples was detected by multiple microspheres flow immunofluorescence luminescence using the certain cytokine detection kit (Qingdao Raisecare Biotechnology Co., Ltd, Shandong Province, China, Cat no.: 20180087). Both the samples were analyzed by fluorescence flow cytometry (DxFLEX, Beckman Coulter, Inc., USA). Subsequent detection procedures were performed following the instructions provided with the respective kits. The detection of immunoglobulin (Ig) and complement in serum was performed by immunoturbidimetry using IMMAGE 800 specific protein analysis system (Beckman Coulter, Inc., USA, Cat no. 2405178), and the relative kits applied to IgA, IgG, IgM, IgE, C3, and C4 were all from Beckman Coulter, Inc., USA (Cat no. 20152400299, 20152400549, 20152400749, 20172402239, 20152400297, and 20152400746). Totally, 41 immune/cytokine indexes were detected in this study, and the list of these indexes is summarized in **Supplementary Table S1**.

### Statistical analysis

Continuous variables were expressed as median and interquartile range (IQR), and the comparison between case and control groups utilized either Student’s t-test or the nonparametric Mann–Whitney U-test. Categorical variables were summarized using counts and percentages and compared by χ^2^ test or Fisher’s exact test. The multivariable analysis to identify the differential urine metabolites was conducted using orthogonal partial least square discriminate analysis (OPLS-DA). Significantly differential metabolites were selected in a criterion of the variable importance in projection (VIP) ≥1.5 and p<0.05. The correlation analysis was performed with Spearman correlation method. The receiver operator characteristic (ROC) curve was constructed, and the area under the curve (AUC) was calculated to assess the discrimination ability between groups.

A random forest (RF) model was constructed to evaluate the importance of lipid super classes, lipids, and lipids combined with cytokines and laboratory indicators in differentiating the severity of COVID-19 patient. The data for different combinations were divided into training and testing sets in an 80:20 ratio for each. The evaluation of the model involved selecting the node value and the number of decision trees that maximize the AUC for each RF model. The mean decrease Gini (MDG) method was implemented to determine the relative importance of factors in distinguishing the severity of COVID-19 patients (20).

The data analysis and visualization were performed using R software (version 4.2.2, R Foundation for Statistical Computing, Vienna, Austria), STATA 17.0 (StataCorp LLC, College Station, TX77845, USA) and with SIMCA (version 14.1, Sartorius Stedim Biotech, Umea, Sweden). A two-sided p<0.05 was considered statistically significant.

## Results

### Patient information

Totally, 44 COVID-19 patients were enrolled in this study, including 16 mild patients and 28 severe patients. The 28 severe patients included 19 survival and nine fatal patients. Among the 19 survival patients in the severe group, there were 15 patients sampled in both acute and recovery stages (**Table 1**). The median (IQR) age of total patients was 71 (65–77) years old, and there were 32 (72.7%) men. No significant differences were observed in all the basic characteristics of mild and severe patients (all p>0.05).

**Table 1:** The characteristics of the COVID-19 patients.

Characteristics	Total (N=44)	Mild (n=16)	Severe (n=28)	P	Survival (n=19)	Fatal (n=9)	P
Age, years, median (IQR)	71 (65–77)	70 (64–75)	72 (69–77)	0.328	78 (77–80)	70 (64–74)	0.002
Sex, male, n (%)	32 (72.7)	14 (87.5)	18 (64.3)	0.083	11 (57.9)	7 (77.8)	0.417
Comorbidity, n (%)	44 (100)	16 (100)	28 (100)	1.000	19 (100)	9 (100)	1.000
Hypertension	28 (63.6)	11 (68.8)	17 (60.7)	0.594	10 (52.6)	7 (77.8)	0.249
Diabetes	18 (40.9)	6 (37.5)	12 (42.9)	0.728	8 (42.1)	4 (44.4)	1.000
Coronary heart disease	10 (22.7)	2 (12.5)	8 (28.6)	0.205	5 (26.3)	3 (33.3)	1.000
Cerebral infarction	8 (18.2)	3 (18.8)	5 (17.9)	0.941	3 (15.8)	2 (22.2)	1.000
Other	21 (47.7)	5 (31.3)	16 (57.1)	0.098	11 (57.9)	5 (55.6)	1.000
General symptoms, n (%)	37 (84.1)	14 (87.5)	23 (82.1)	0.635	15 (78.9)	8 (88.9)	1.000
Fever	32 (72.7)	12 (75)	20 (71.4)	0.797	14 (73.7)	6 (66.7)	1.000
Chill	1 (2.3)	0 (0)	1 (3.6)	1.000	0 (0)	1 (11.1)	0.321
Headache	2 (4.5)	0 (0)	2 (7.1)	0.526	2 (10.5)	0 (0)	1.000
Dizzy	1 (2.3)	0 (0)	1 (3.6)	1.000	1 (5.3)	0 (0)	1.000
Feeble	9 (20.5)	4 (25)	5 (17.9)	0.576	3 (15.8)	2 (22.2)	1.000
Muscular soreness	6 (13.6)	4 (25)	2 (7.1)	0.104	2 (10.5)	0 (0)	1.000
Respiratory symptoms, n (%)	42 (95.5)	15 (93.8)	27 (96.4)	1.000	18 (94.7)	9 (100)	1.000
Cough	35 (79.5)	11 (68.8)	24 (85.7)	0.187	16 (84.2)	8 (88.9)	1.000
Expectoration	30 (68.2)	10 (62.5)	20 (71.4)	0.613	14 (73.7)	6 (66.7)	1.000
Dyspnea	30 (68.2)	8 (50)	22 (78.6)	0.051	13 (68.4)	9 (100)	0.136
Rhinorrhea	1 (2.3)	1 (6.3)	0 (0)	0.364	0 (0)	0 (0)	–
Asthma	5 (11.4)	1 (6.3)	4 (14.3)	0.377	2 (10.5)	2 (22.2)	0.582
Anhelation	8 (18.2)	2 (12.5)	6 (21.4)	0.418	2 (10.5)	4 (44.4)	0.136

COVID-19, coronavirus disease 2019; IQR, interquartile range.

### Lipidome detection results

**Figure 1:**
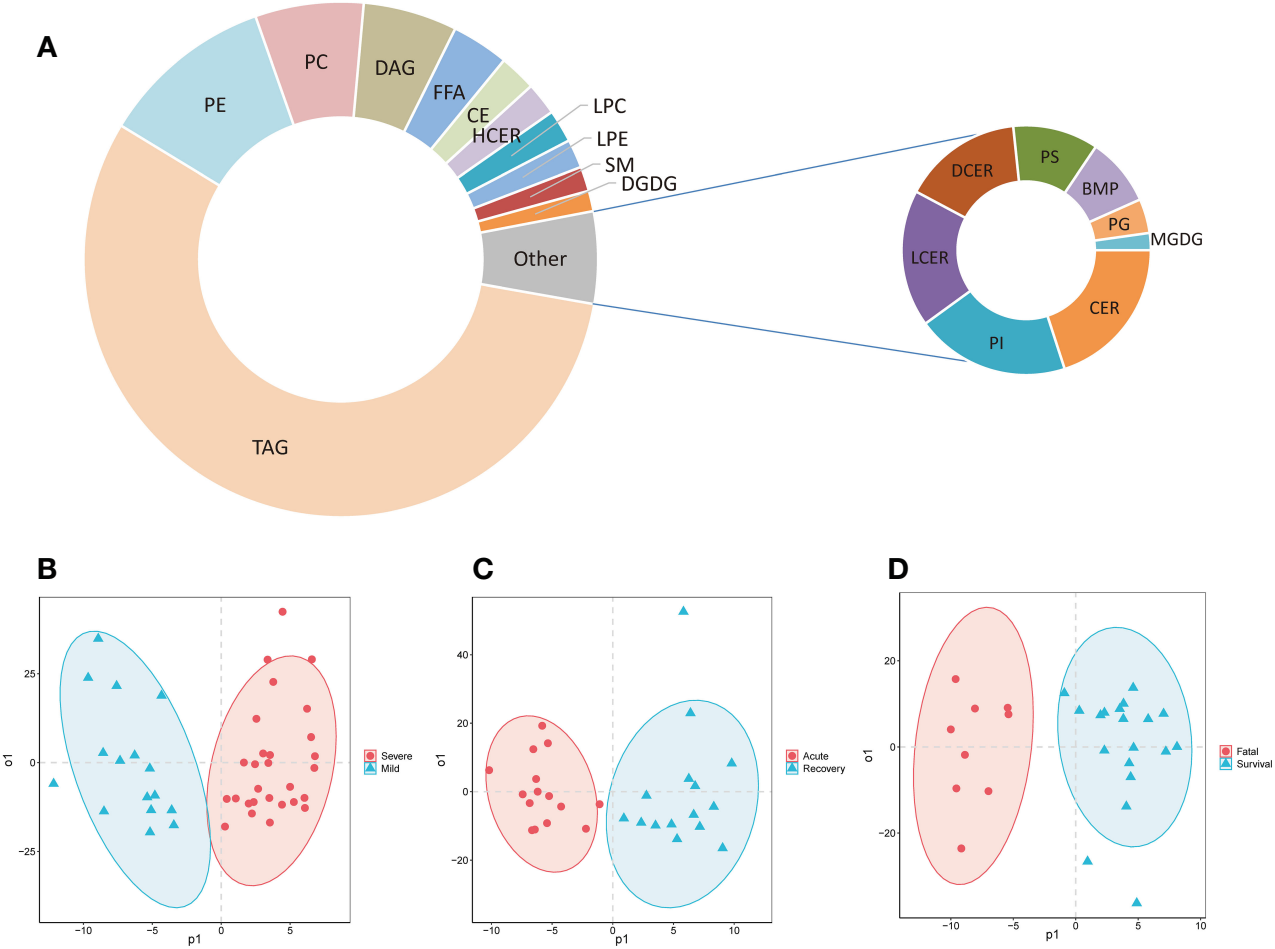
The composition of detected lipids and OPLS-DA models. **(A)** The doughnut chart of detected lipids; **(B)** the OPLS-DA model of mild and severe patients; **(C)** the OPLS-DA model of severe patients in acute and recovery stages; **(D)** the OPLS-DA model of survival and fatal patients. FFA, free fatty acids; MGDG, monogalactosyldiacylglycerol; DGDG, digalactosyldiacylglycerol; DAG, diacylglycerols; TAG, triacylglycerols; CE, cholesterol esters; CER, ceramides; DCER, dihydroceramides; HCER, hexosylceramides; LCER, lactosylceramides; SM, sphingomyelins; LPC, lysophosphatidylcholines; LPE, lysophosphatidylethanolamines; LPG, lyso-phosphatidylglycerol; LPI, lyso-phosphatidylinositol; LPS, lyso-phosphatidylserine; PC, phosphatidylcholines; PE, phosphatidylethanolamines; PG, phosphatidylglycerol; PI, phosphatidylinositol; PS, phosphatidylserine; PIP, phosphatidylinositol phosphate; BMP, bis(monoacylglycero)phosphate; OPLS-DA, orthogonal partial least squares-discriminant analysis.

Totally, 778 lipids were detected and classified into 19 super classes from the serum of COVID-19 patients (**Figure 1A**; **Supplementary Table S2**). The top 5 super classes were triacylglycerols (TAGs, 435, 55.9%), phosphatidylethanolamines (PEs, 85, 10.9%), phosphatidylcholines (PCs, 53, 6.8%), diacylglycerols (DAGs, 46, 5.9%), and free fatty acids (FFAs, 28, 3.6%). The OPLS-DA models showed that these lipids separated mild and severe patients, acute and recovery states patients, and survival and fatal patients, respectively (**Figures 1B–D**).

### Differential lipid metabolites

**Figure 2:**
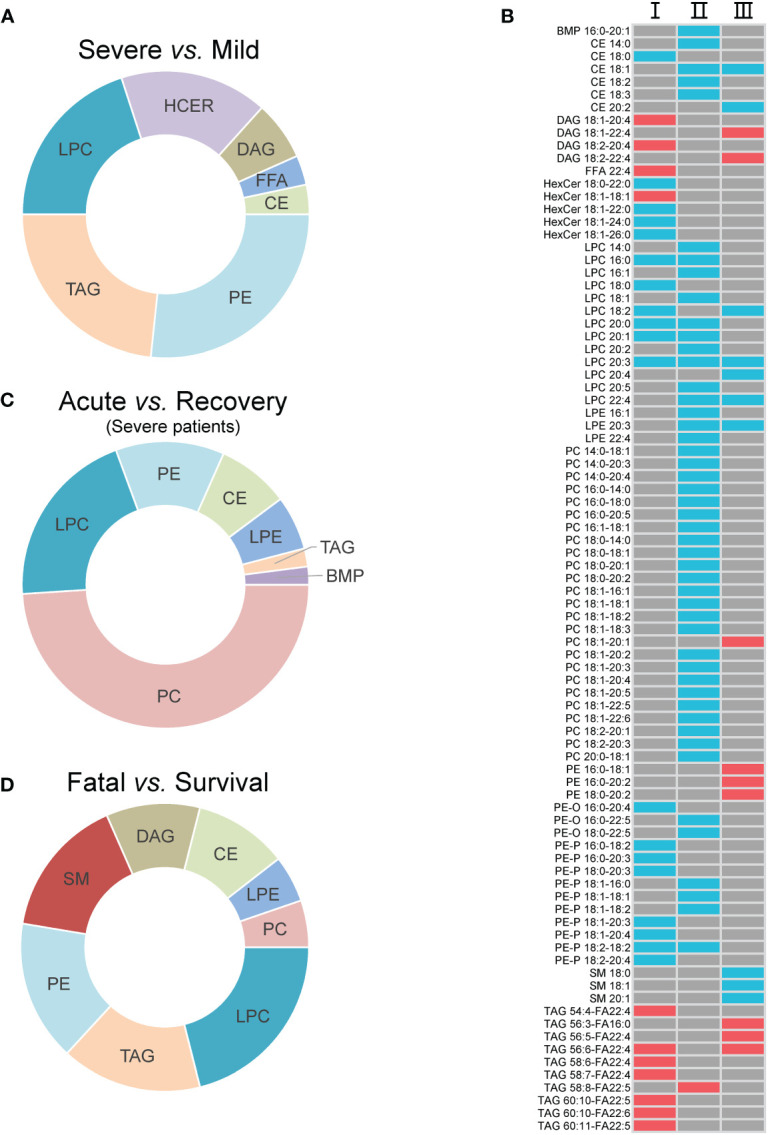
The inter-group differential lipids and their alteration. **(A)** The super classes of differential lipids between mild and severe patients; **(B)** the alteration of the differential lipids between groups. I, II, and III refer to severe vs. mild, acute vs. recovery, and fatal vs. survival, respectively; **(C)** the super classes of differential lipids between severe patients in acute and recovery stages; **(D)** the super classes of differential lipids between survival and fatal patients. The red cell means upregulation, and the blue cell means downregulation. The gray cell means that there was no significant difference between two groups. FFA, free fatty acids; MGDG, monogalactosyldiacylglycerol; DAG, diacylglycerols; TAG, triacylglycerols; CE cholesterol esters; HCER, hexosylceramides; SM, sphingomyelins; LPC, lysophosphatidylcholines; LPE, lysophosphatidylethanolamines; PC, phosphatidylcholines; PE, phosphatidylethanolamines; BMP, bis(monoacylglycero)phosphate.

Seven super classes with 30 differential lipid metabolites were observed between mild and severe patients, including PEs (8, 26.7%), TAGs (7, 23.3%), lysophosphatidylcholines (LPCs, 6, 20.0%), hexosylceramides (HCERs, 5, 16.7%), DAGs (2, 6.7%), FFAs (1, 3.3%), and CEs (1, 3.3%) (**Figure 2A**; **Supplementary Table S3**). Among all the 30 differential lipid metabolites, those of LPCs, CEs and PEs, and HCERs except for HexCer 18:1-18:1 were significantly lower in severe patients compared to mild patients, while all the metabolites of TAGs and DAGs, and HexCer 18:1-18:1 were significantly higher in severe patients compared to mild patients (**Figure 2B**). There were 49 differential metabolites between the acute and recovery stages, categorized as PCs (24, 39.0%), LPCs (4, 22.2%), PEs (3, 16.7%), CEs (4, 18.2%), lysophosphatidylethanolamines (LPEs, 3, 6.1%), TAGs (1, 2.0%), and bis(monoacylglycero)phosphates (1, 2.0%) (**Figure 2C**; **Supplementary Table S3**). The concentration of all these 49 differential lipid metabolites except for TAG 58:8-FA22:5 was significantly lower in the acute stage of severe patients compared to the recovery stage (**Figure 2B**). Between survival and fatal patients, 19 lipids were significantly different, consisting of eight super classes including LPCs (4, 22.2%), TAGs (3, 16.7%), PEs (3, 16.7%), sphingomyelins (SMs, 3, 16.7%), DAGs (2, 11.1%), CEs (2, 11.1%), LPEs (1, 5.3%), and PCs (1, 5.3%) (**Figure 2D**; **Supplementary Table S3**). The concentration of these 19 differential lipid metabolites in CEs, LPCs, LPEs, and SMs was significantly lower in fatal patients compared to survival patients, while it was opposite for DAGs, PCs, Pes, and TAGs (**Figure 2B**).

### Correlation of lipids and immune/cytokine indexes

**Figure 3:**
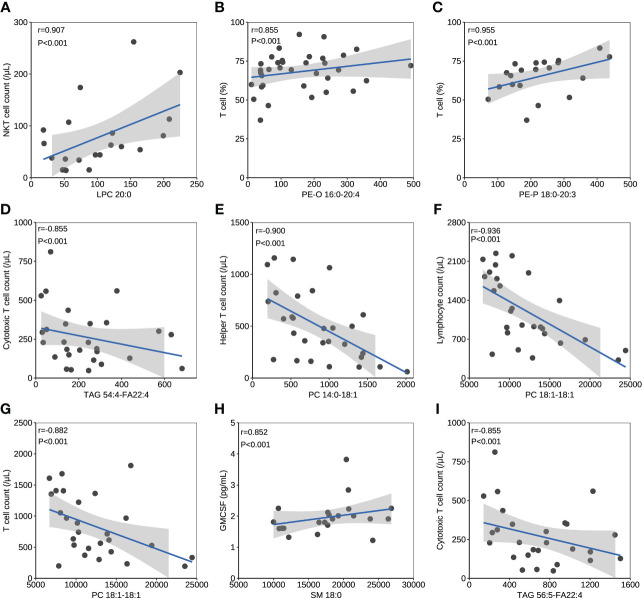
The correlation between lipids and immune/cytokine indexes. **(A)** The correlation between levels of LPC 20:0 and NKT cell count. **(B)** The correlation between levels of PE-O 16:0-20:4 and T cell percentage. **(C)** The correlation between levels of PE-P 18:0-20:3 and T cell percentage. **(D)** The correlation between levels of TAG 54:4-FA22:4 and cytotoxic T cell count. **(E)** The correlation between levels of PC 14:0-18:1 and helper T cell count. **(F)** The correlation between levels of PC 18:1-18:1 and lymphocyte count. **(G)** The correlation between levels of PC 18:1-18:1 and T cell count. **(H)** The correlation between levels of SM 18:0 and GMCSF. **(I)** The correlation between levels of TAG 56:5-FA22:4 and cytotoxic T cell count. Datapoints are index values. The line is the fitted curve and the shadow is its 95% confidence interval. NKT cell, natural killer T cell; GMCSF, granulocyte-macrophage colony-stimulating factor.

The correlation between lipids and immune/cytokine indexes were explored, and the correlation coefficient >0.85 was selected. Four differential lipids between mild and severe patients turned to have significant correlations with certain immune/cytokine indexes. Significantly positive correlation was observed between LPC 20:0 and natural killer T (NKT) cell count, PE-O 16:0-20:4 and T cell percentage, and PE-P 18:0-20:3 and T cell percentage, while the negative correlation was observed between TAG 54:4-FA22:4 and cytotoxic T cell count (**Figures 3A–D**). PC 14:0-18:1 and helper T-cell count, PC 18:1-18:1 and lymphocyte count, and PC 18:1-18:1 and T-cell count were detected to be negatively correlated, and these three lipids were different between the acute and recovery stages in severe patients (**Figures 3E–G**). Two differential lipids between survival and fatal patients turned to be significantly correlated with granulocyte-macrophage colony-stimulating factor (GMCSF) or cytotoxic T-cell count. Significantly positive correlation was observed between SM 18:0 and GMCSF, while negative correlation was observed between TAG 56:5-FA22:4 and cytotoxic T-cell count (**Figures 3H, I**).

### Alteration and discrimination of the key lipids

**Figure 4:**
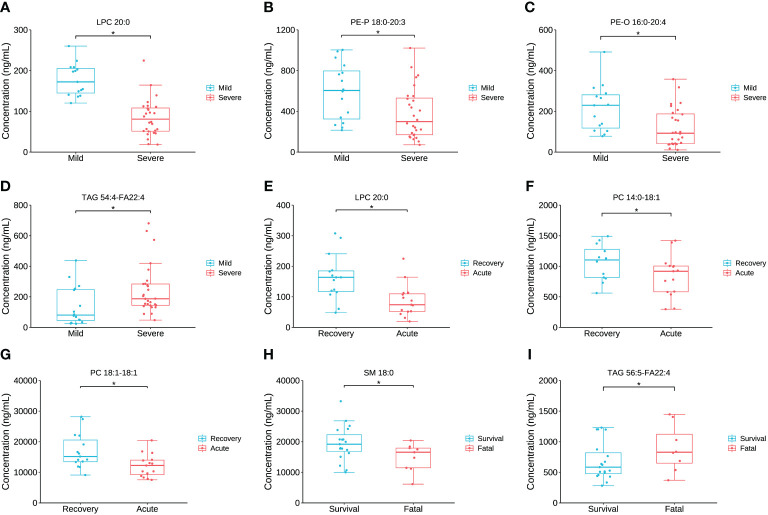
The inter-group comparison of the concentration of the key lipids between groups. **(A)** The concentration comparison of LPC 20:0 between severe and mild groups. **(B)** The concentration comparison of PE-P 18:0-20:3 between severe and mild groups. **(C)** The concentration comparison of PE-O 16:0-20:4 between severe and mild groups. **(D)** The concentration comparison of TAG 54:4-FA22:4 between severe and mild groups. **(E)** The concentration comparison of LPC 20:0 between acute and recovery stages of severe patients. **(F)** The concentration comparison of PC 15:0-18:1 between acute and recovery stages of severe patients. **(G)** The concentration comparison of PC 18:1-18:1 between acute and recovery stages of severe patients. **(H)** The concentration comparison of SM 18:0 between fatal and survival groups. **(I)** The concentration comparison of TAG 56:5-FA22:4 between fatal and survival groups. Datapoints are index values, the middle line refers to the median of each group and error bars show inter-quartile range. *P<0.05.

**Figure 5:**
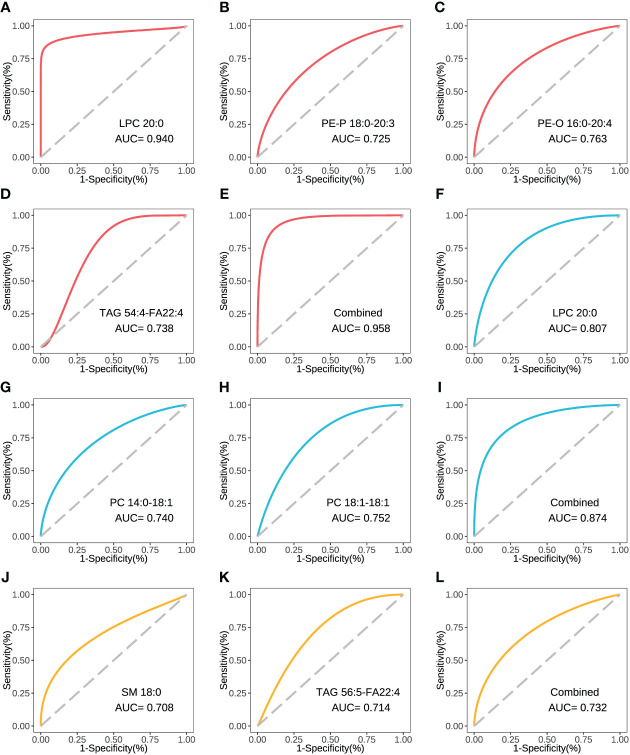
The ROC curve of the key inter-group differential lipids. **(A)** The ROC and AUC of LPC 20:0 in severe and mild groups. **(B)** The ROC and AUC of PE-P 18:0-20:3 in severe and mild groups. **(C)** The ROC and AUC of PE-O 16:0-20:4 in severe and mild groups. **(D)** The ROC and AUC of TAG 54:4-FA22:4 in severe and mild groups. **(E)** The combined ROC and AUC of the above 4 lipids in severe and mild groups. **(F)** The ROC and AUC of LPC 20:0 in acute and recovery stages in severe patients. **(G)** The ROC and AUC of PC 14:0-18:1 in acute and recovery stages in severe patients. **(H)** The ROC and AUC of PC 18:1-18:1 in acute and recovery stages in severe patients. **(I)** The combined ROC and AUC of the above 3 lipids in acute and recovery stages in severe patients. **(J)** The ROC and AUC of SM 18:0 in fatal and survival groups. **(K)** The ROC and AUC of TAG 56:5-FA22:4 in fatal and survival groups. **(L)** The combined ROC and AUC of the above 2 lipids in fatal and survival groups. The plots with red line refer to the lipids between severe and mild patients. The plots with blue line refer to the lipids between severe patients in acute and recovery stages. The plots with yellow line refer to the lipids between fatal and survival patients. ROC, receiver operator characteristic.

The concentration comparison of the lipid metabolites related to immune/cytokine indexes above between groups is displayed in **Figure 4**. The concentrations of LPC 20:0, PE-P 18:0-20:3, and PE-O 16:0-20:4 were significantly lower in severe patients compared to that in mild patients, while the opposite was true for TAG 54:4-FA22:4 (**Figures 4A–C**). The concentrations of LPC 20:0, PC 14:0-18:1, and PC 18:1-18:1 were significantly lower in the acute stage of severe patients than that in the recovery stage (**Figures 4F, G**). For the comparison between the fatal and survival patients, the concentration of SM 18:0 was significantly lower, while TAG 56:5-FA22:4 was significantly higher (**Figures 4H, I**). The ROC showed the abilities of these metabolites in discriminating the corresponding two groups, and the AUC of LPC 20:0 reached 0.940 in discriminating mild and severe patients (**Figure 5A**). Between mild and severe patients, the AUCs of the PE-P 18:0-20:3, PE-O 16:0-20:4, and TAG 54:4-FA22:4 were 0.725, 0.763, and 0.738, respectively (**Figures 5B–D**). The AUC of all the differential lipids combined between the two groups reached 0.958 (**Figure 5E**). Between the acute and recovery stages in severe patients, the AUCs of the LPC 20:0, PC 14:0-18:1, and PC 18:1-18:1 were 0.807, 0.740, and 0.752, respectively, and 0.874 for the three combination (**Figures 5F–I**). Between survival and fatal patients, the AUCs of SM 18:0 and TAG 56:5-FA22:4 were 0.708 and 0.714, respectively, and 0.732 for the combination (**Figures 5J–L**).

### Immune/cytokine differences between patients with high and low level of LPC 20:0

**Figure 6:**
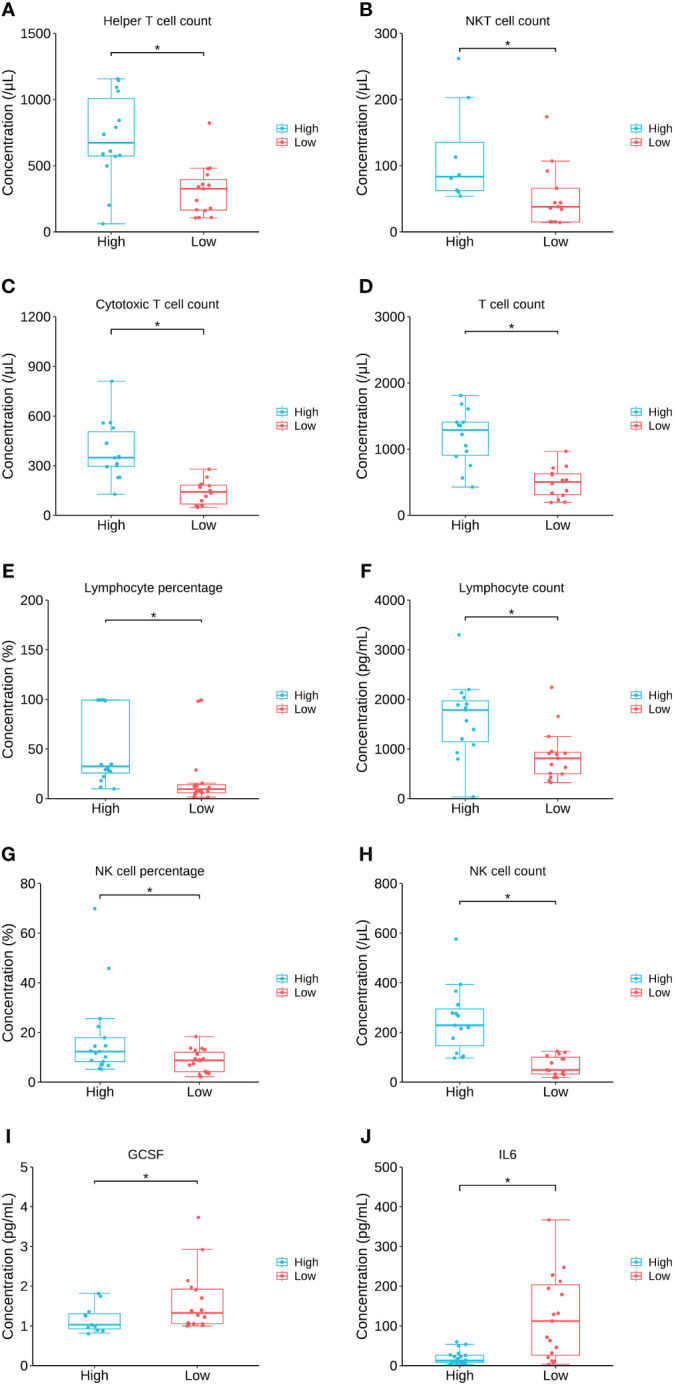
The comparison of immune/cytokine indexes between patients with high and low levels of LPC 20:0. **(A–J)** are the comparisons of helper T cell count, NKT cell count, cytotoxic Tcell count, T cell count, lymphocyte percentage, lymphocyte count, NK cell percentage, NK cell count, GCSF and IL6 between patients with high and low LPC 20:0. Datapoints are index values, the middle line refers to the median of each group and error bars show inter-quartile range. NKT cell, natural killer T cell; GCSF, granulocyte colony stimulating factor; IL6, interleukin 6. *P<0.05.

To further investigate the relation between the key lipid LPC 20:0 and immune/cytokine indexes, those 44 patients were divided into high LPC 20:0 and low LPC 20:0 groups according to its median concentration, and then inter-group differences of immune/cytokine indexes were explored. The level of helper T-cell count, NKT cell count, cytotoxic T-cell count, T-cell count, lymphocyte count/percentage, and NK cell count/percentage were all significantly lower in the low LPC 20:0 group than in high LPC 20:0 group (all p<0.05, **Figures 6A–H**; **Supplementary Table S4**). For cytokines, the concentration of granulocyte colony stimulating factor (GCSF) and interleukin 6 (IL-6) both significantly increased in the low LPC 20:0 group (both p<0.05, **Figures 6I, J**; **Supplementary Table S4**). The other immune/cytokine indexes investigated in this study were not significantly different between the two groups (all p>0.05, **Supplementary Table S4**).

### Lipids importance identified by the RF model

**Figure 7:**
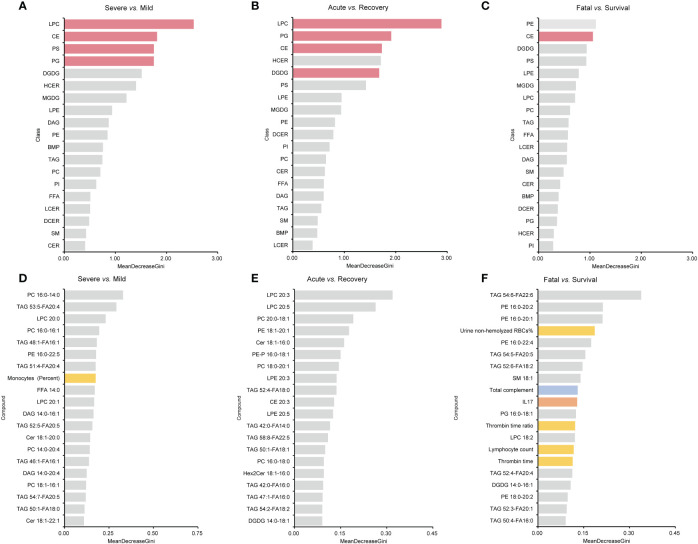
MDG top ranking lipid super classes, lipids, and all the indicators generated by RF models. **(A–C)** are the results of lipid super classes, and columns in red present lipids super classes with significance. **(D–F)** are the results of all the indicators. Columns in blue present indicators belonging to immune indexes. Columns in orange present indicators belonging to cytokines. Columns in yellow present indicators belonging to laboratory indicators. MDG, mean decrease Gini; RF, random forest.

For lipid super classes, the relatively significant importance of CE was confirmed by the RF model in distinguishing the three groups of COVID-19 patients, while LPCs showed a high importance in both the mild/severe groups and the acute/recovery stages (**Figures 7A–C**). When analyzing lipids in combination with one of the indicators, only a few indicators other than lipids showed a high importance (**Supplementary Figure S1**). Immunoglobulin M ranked 14th in combination of lipids and immune cells in distinguishing the acute/recovery stages (**Supplementary Figure S1E**). The importance of IL-17 was relatively forward in distinguishing death from survival patients among combinations of lipids and cytokines (**Supplementary Figure S1I**). In the RF model combining lipids and laboratory indicators, laboratory indicators showed a certain degree of importance in differentiating three groups (**Supplementary Figures S1J–L**). The MDG of the RF model established by combining lipids with immune cells, cytokines, laboratory indicators, and clinical systems showed that lipids were the primary indicators to distinguish patient types and clinical stages (**Figures 7D–F**). For the above RF models, their AUCs had reached 0.833 or above.

## Discussion

In this study, we summarized the lipid metabolic profiles in patients with mild or severe COVID-19 patients, indicating that certain lipids were associated with the immune/inflammatory responses and the progression and severity of the disease. LPC 20:0 turned to have the potential in differentiating mild/severe patients and acute/recovery stages of severe patients. These findings suggested the underlying importance of lipids, especially LPCs, in predicting the progression and outcome, and identifying potential treatment targets of COVID-19 patients.

Metabolomic and lipidomic approaches have elucidated numerous circulating lipids that are directly associated with the severity of COVID-19, indicating that lipid metabolism could be a potential target for therapeutic strategies in the treatment and management of the disease (21, 22). Circulating lipids play a key function in the pathogenesis of SARS-CoV-2. A deeper understanding of lipid metabolism in host–pathogen interactions will offer valuable insights into viral pathogenesis and the development of novel therapeutic targets. The study results further highlighted the importance of lipidome in predicting the advancement and seriousness of COVID-19 using RF models. The correlation between lipids and immune/cytokine markers could provide valuable insights into how the lipidome impacts disease progression. Particularly, LPC 20:0 appeared to play a significant role in the predictive analysis, as shown by the RF models and AUC.

As bioactive lipid metabolites of phosphatidylcholines (PCs), plasma lysophosphatidylcholines (LPCs) are primarily generated by the action of secretory phospholipases A2 (sPLA2) following the removal of a fatty acid (23). In a recent study by Lin et al., it was shown that LPCs in plasma can inhibit the production of reactive oxygen species (ROS) and the activation of neutrophils, showcasing the anti-inflammatory effects of LPCs in an *ex vivo* lung perfusion model (24). Several recent studies have reported that lower levels of plasma LPCs were remarkably associated with unfavorable disease outcomes. Reduced LPC concentrations have been observed in connection with an increased risk of mortality in conditions such as diabetes, asthma, schizophrenia, Alzheimer’s disease, polycystic ovary syndrome, pulmonary arterial hypertension, rheumatoid arthritis, liver cirrhosis, and aging (25). In our study, a notable decrease in LPCs was observed in patients with severe COVID-19, especially in those in the acute stage and those with fatal outcome, consistent with other studies (2, 18). LPC 20:0, one of LPCs, turned to be correlated with disease severity and showed a high AUC in distinguish the severity of COVID-19. Consistently, another study also highlighted LPC 20:0 as an important lipid in classifying the severity of COVID-19 (26). In addition, we discovered that the decreased level of LPC 20:0 exhibited a correlation with the reduction in NKT cell count, and the concentrations of GCSF and IL-6 decreased in the lower LPC 20:0 group. These findings further suggested that SARS-CoV-2 might have a significant impact on the metabolism of phospholipids containing unsaturated fatty acyl groups (2).

Those decreased LPCs containing unsaturated acyl groups such as LPC 20:0 might impact the host’s immune or inflammatory responses by affecting the interaction with NKT cells, GCSF, and IL-6, and then lead to unfavorable outcomes. A previous study has reported that LPC-mediated activation of type II NKT cells could induce anergy induction in type I NKT cells and help protect against Con A-induced hepatitis, shedding light on the relationship between LPCs and NKT cells in SARS-CoV-2 infection (27). In addition, similar to the result that, overall, a higher IL-6 concentration was associated with a lower LPC 20:0 in our study, a recent study reported the concentration of LPCs was negatively linked to the severity of community-acquired pneumonia. This correlation could inhibit the secretion of IL-1β, IL-6, and TNF-α, and the production of ROS, and reduce the depletion of superoxide dismutase and glutathione, thereby reducing lung tissue and cell damage and retarding the disease progression (28).

In our study, PCs were significantly reduced in the acute stage of severe patients and showed an overall sustained reduction over the disease course of fatal COVID-19 cases. Those findings indicated that PCs might alter mainly during the active response process of the host against the virus infection (29). As a dominant component of biological membrane, PCs act as structural and regulative roles in signal transduction, immune activation pathways, and inflammatory responses. Earlier studies have shown the PCs are involved in in the formation of the immunological synapse, the activation of macrophages, and the differentiation and functioning of T and B effector cells (30). The negative correlations between PCs and helper T-cell count, lymphocyte count, and T cell between mild/severe COVID-19 patients suggested a worse immune state during disease progression.

In addition, an upregulation of TAGs in patients with severe COVID-19 was observed, especially in those in acute stage and with fatal outcome. DAGs were also elevated in severe patients, with notably overall higher concentrations in fatal patients compared to those who survived. However, no significant difference in DAG levels was observed between the acute and recovery stages of the disease. In addition, a significant correlation was observed between the decrease in TAGs and the increase in cytotoxic T-cell count here, further suggesting the harmful effects of TAGs reduction. Wu et al. also reported that the levels of DAGs and TAGs were higher in the fatal COVID-19 cases (31), consistent with our finding. But in another study, TAGs were significantly lower in mild patients (32). Actually, lipolysis of adipose tissue enhances resulting from infection, converting TAG to non-esterified fatty acids (NEFAs) and DAG, and also strengthening the recycling of fatty acids back into TAGs (21). Thus, the alteration of DAGs and TAGs might be floating in different stages of the infection course.

We observed the reduced levels of SMs in fatal patients compared to survival patients. A study conducted by Shen et al. revealed that the levels of serum SMs were also reduced in both severe and non-severe COVID-19 patients with (33). Enhanced levels of SMs in COVID-19 patients has also been observed (32). SMs are the major sphingolipids in mammalian cell membranes and contribute a lot in plenty of cell biological processes, including the regulation of endocytosis, ion channels, and protein sorting (34, 35). Studies have shown that SMs play an important role in virus entry, receptor segregation and pathogen sorting, endocytosis, virus replication, and virus maturation (36). Thus, the reduction in SMs might suggest fatal virus invasion and corrupted dysfunction of host cells. In our study, the decreased level of SM 18:0 showed a correlation with a reduction in GMCSF. However, GMCSF is known to contribute to the cytokine storm, which can worsen the severity of COVID-19 and potentially lead to fatal outcome in patients (37). The contradict result might attribute to the bias resulting from lack of data.

There were several limitations of this study. First, we did not perform a comprehensive detection of all lipids, but we conducted targeted metabonomic analysis for the main lipids associated with viral infection, which could describe the main lipid profile as well. Second, the relatively small sample size utilized in this study could introduce potential biases to the results. Third, this study only focused on COVID-19 patients with pneumonia and did not include patients who only showed upper respiratory tract infection, which may have certain limitations in extrapolation. Finally, the lack of comparison with a control group limited the understanding of the mechanism of COVID-19, and further research for the specific role of lipidome or lipid metabolism in COVID-19 and other respiratory viral infection is needed.

In conclusion, the lipidome was associated with the progression of COVID-19. Lipids might play a role in immune responses and serve as important indicators for predicting outcomes. LPC 20:0 might be a potential predictive target in the progression and outcome and the possible treatment target of COVID-19 patients. These findings provided new evidence to the importance of lipids in understanding COVID-19.

## Data availability statement

The raw data supporting the conclusions of this article will be made available by the authors, without undue reservation.

## Ethics statement

The studies involving humans were approved by Peking University Third Hospital Medical Science Research Ethics Committee (2023–014–02). The studies were conducted in accordance with the local legislation and institutional requirements. The participants provided their written informed consent to participate in this study.

## Author contributions

SZ: Data curation, Formal analysis, Methodology, Software, Visualization, Writing – original draft. ZZ: Data curation, Funding acquisition, Investigation, Supervision, Writing – original draft. WZ: Formal analysis, Investigation, Visualization, Writing – original draft. RW: Data curation, Investigation, Writing – review & editing. FL: Data curation, Investigation, Writing – review & editing. HY: Formal analysis, Writing – review & editing. QZ: Data curation, Investigation, Writing – review & editing. TW: Formal analysis, Writing – review & editing. JX: Data curation, Investigation, Writing – review & editing. YZ: Supervision, Writing – review & editing. TW: Investigation, Writing – review & editing. JD: Formal analysis, Writing – review & editing. NH: Supervision, Writing – review & editing. QG: Conceptualization, Funding acquisition, Supervision, Writing – review & editing. QL: Conceptualization, Methodology, Supervision, Writing – review & editing.

## Supplementary material

Supplementary Figure 1 MDG top ranking lipids combined with other indicators generated by RF models. **(A)** lipids; **(B)** lipids and immune indexes; **(C)** lipids and cytokines; **(D)** lipids and laboratory indicators; **(E)** lipids and clinical symptoms. Columns in blue presented indicators belonging to immune indexes. Columns in orange presented indicators belonging to cytokines. Columns in yellow presented indicators belonging to laboratory indicators. MDG, mean decrease Gini; RF, random forest.
