# Supplementary figures and images for: Lipidome is a valuable tool for the severity prediction of coronavirus disease 2019
Source: Front Immunol. 2024 May 10;15:1337208. doi: 10.3389/fimmu.2024.1337208 (PMC11116732; doi:10.3389/fimmu.2024.1337208)

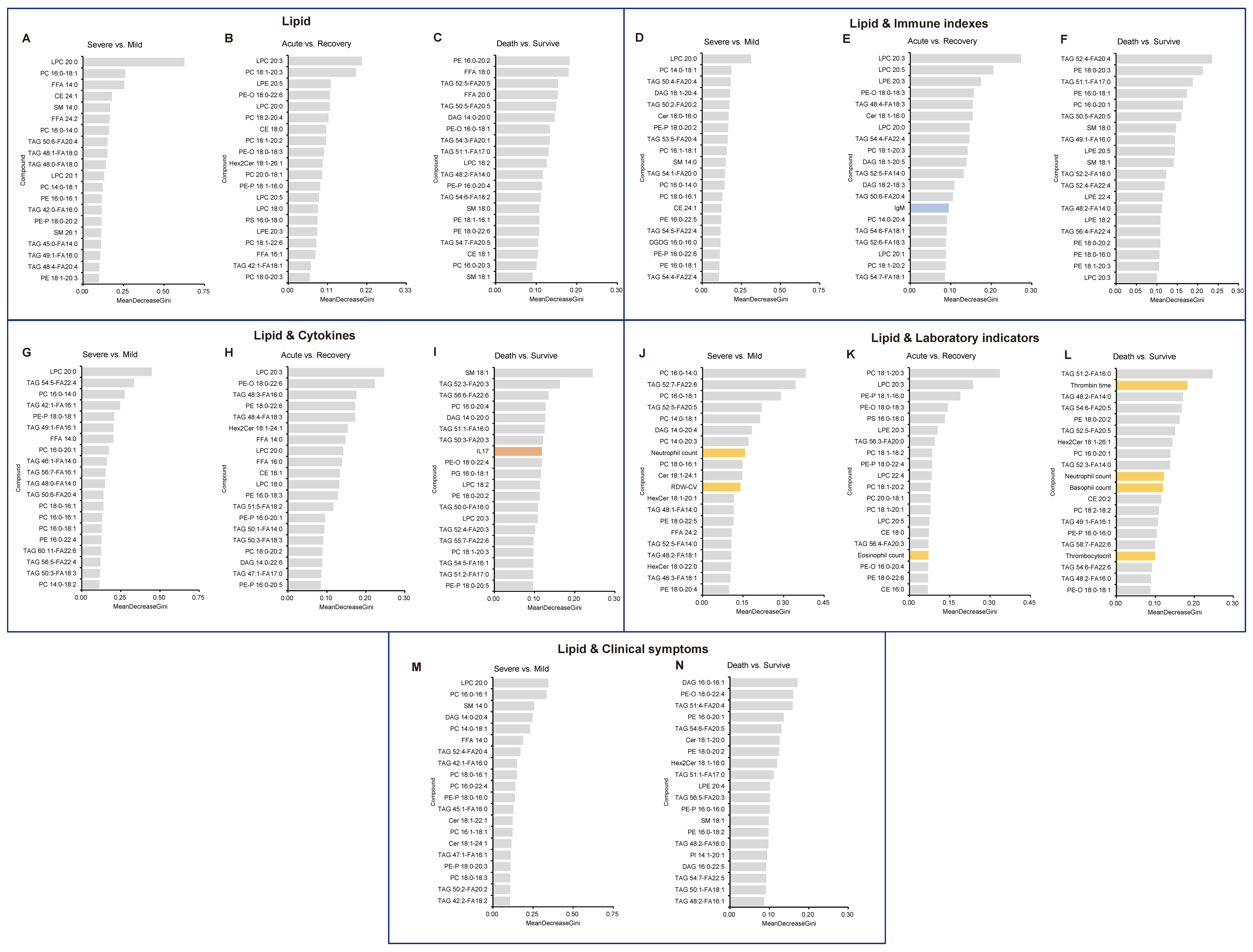

Supplement: Supplementary Figure 1 — MDG top ranking lipids combined with other indicators generated by RF models. (A) lipids; (B) lipids and immune indexes; (C) lipids and cytokines; (D) lipids and laboratory indicators; (E) lipids and clinical symptoms. Columns in blue presented indicators belonging to immune indexes. Columns in orange presented indicators belonging to cytokines. Columns in yellow presented indicators belonging to laboratory indicators. MDG, mean decrease Gini; RF, random forest. [file Image_1.tif]
