# Supplementary material for: Lipidome is a valuable tool for the severity prediction of coronavirus disease 2019
Source: Front Immunol. 2024 May 10;15:1337208. doi: 10.3389/fimmu.2024.1337208 (PMC11116732; doi:10.3389/fimmu.2024.1337208)
Supplement: Supplementary file 2 [file DataSheet_1.docx]

**Supplementary Table 1. The list of immune/cytokine indexes detected in this study**

| **Indexes** | **Unit** |
| --- | --- |
| T cell percentage | % |
| Helper T cell percentage | % |
| Cytotoxic T cell percentage | % |
| Lymphocyte percentage | % |
| Natural killer lymphocyte percentage | % |
| Natural killer T cell percentage | % |
| B lymphocyte percentage | % |
| Regulatory T cell percentage | % |
| T cell count | /μL |
| Helper T cell count | /μL |
| Cytotoxic T cell count | /μL |
| Lymphocyte count | /μL |
| Natural killer lymphocyte count | /μL |
| Natural killer T cell count | /μL |
| B lymphocyte count | /μL |
| Th/Tc (Helper T cell/ Cytotoxic T cell ) | – |
| IgG | g/L |
| IgA | g/L |
| IgM | g/L |
| IgE | g/L |
| C3 | g/L |
| C4 | g/L |
| Interleukin-1β | pg/mL |
| Interleukin-2 | pg/mL |
| Interleukin-4 | pg/mL |
| Interleukin-5 | pg/mL |
| Interleukin-6 | pg/mL |
| Interleukin-8 | pg/mL |
| Interleukin-9 | pg/mL |
| Interleukin-10 | pg/mL |
| Interleukin-12P70 | pg/mL |
| Interleukin-13 | pg/mL |
| Interleukin-17 | pg/mL |
| Interferon α | pg/mL |
| Interferon γ | pg/mL |
| Tumor necrosis factor α | pg/mL |
| Granulocyte colony stimulating factor | pg/mL |
| Granulocyte macrophage colony stimulating factor | pg/mL |
| Vascular endothelial growth factor | pg/mL |
| Macrophage inflammatory protein 1α | pg/mL |
| Monocyte chemoattractant protein 1 | pg/mL |

**Supplementary Table 2. The composition of lipid metabolites**

| **Super Class** | **Number** | **Percentage (%)** |
| --- | --- | --- |
| TAG | 435 | 55.9 |
| PE | 85 | 10.9 |
| PC | 53 | 6.8 |
| DAG | 46 | 5.9 |
| FFA | 28 | 3.6 |
| CE | 18 | 2.3 |
| HCER | 16 | 2.1 |
| LPC | 16 | 2.1 |
| LPE | 14 | 1.8 |
| SM | 12 | 1.5 |
| DGDG | 10 | 1.3 |
| CER | 9 | 1.2 |
| PI | 9 | 1.2 |
| LCER | 8 | 1.0 |
| DCER | 7 | 0.9 |
| PS | 5 | 0.6 |
| BMP | 4 | 0.5 |
| PG | 2 | 0.3 |
| MGDG | 1 | 0.1 |

**Supplementary Table 3. The composition of inter–group differential lipid metabolites**

| **Super Class** | **Number** | **Percentage (%)** |
| --- | --- | --- |
| Severe *vs.* mild | 30 |  |
| PE | 8 | 26.7 |
| TAG | 7 | 23.3 |
| LPC | 6 | 20.0 |
| HCER | 5 | 16.7 |
| DAG | 2 | 6.7 |
| FFA | 1 | 3.3 |
| CE | 1 | 3.3 |
| Acute *vs.* recovery (Severe patients) | 49 |  |
| PC | 24 | 49 |
| LPC | 10 | 20.4 |
| PE | 6 | 12.2 |
| CE | 4 | 8.2 |
| LPE | 3 | 6.1 |
| TAG | 1 | 2.0 |
| BMP | 1 | 2.0 |
| Fatal *vs.* survival | 19 |  |
| LPC | 4 | 22.2 |
| TAG | 3 | 16.7 |
| PE | 3 | 16.7 |
| SM | 3 | 16.7 |
| DAG | 2 | 11.1 |
| CE | 2 | 11.1 |
| LPE | 1 | 5.3 |
| PC | 1 | 5.3 |

**Supplementary Table 4. The immune/cytokine indexes between patients with high LPC 20:0 low LPC 20:0**

| **Immune/Cytokine indexes** | **Total** | **High LPC 20:0** | **Low LPC 20:0** | **P** |
| --- | --- | --- | --- | --- |
| T cell (%) | 325.94 (164.00–395.50) | 72.90 (65.03–74.55) | 66.54 (59.10–74.11) | 0.265 |
| Helper T cell (%) | 66.54 (59.10–74.11) | 40.40 (35.57–54.21) | 37.15 (32.72–44.96) | 0.170 |
| Cytotoxic T cell (%) | 37.15 (32.72–44.96) | 23.43 (15.52–29.10) | 17.74 (13.35–27.61) | 0.324 |
| Lymphocyte (%) | 17.74 (13.35–27.61) | 32.51 (25.88–99.31) | 9.62 (5.81–14.12) | <0.001 |
| Natural killer lymphocyte (%) | 9.62 (5.81–14.12) | 12.31 (8.23–17.90) | 8.72 (4.12–12.03) | 0.038 |
| Natural killer T cell (%) | 8.72 (4.12–12.03) | 5.93 (4.63–6.30) | 4.82 (3.25–8.88) | 0.651 |
| B lymphocyte (%) | 4.82 (3.25–8.88) | 10.31 (6.80–17.33) | 21.88 (7.64–28.56) | 0.145 |
| Regulatory T cell (%) | 21.88 (7.64–28.56) | 6.92 (6.28–7.85) | 5.25 (4.19–8.29) | 0.266 |
| T cell count (/μL) | 5.25 (4.19–8.29) | 1289.24 (908.53–1408.50) | 504.65 (310.00–629.25) | <0.001 |
| Helper T cell count (/μL) | 504.65 (310.00–629.25) | 673.31 (572.75–1007.77) | 325.94 (164.00–395.50) | 0.001 |
| Cytotoxic T cell count (/μL) | 325.94 (164.00–395.50) | 348.63 (295.75–505.63) | 149.00 (74.50–186.18) | 0.001 |
| Lymphocyte count (/μL) | 149.00 (74.50–186.18) | 1787.00 (1145.55–1970.00) | 814.00 (500.63–935.00) | 0.004 |
| Natural killer lymphocyte count (/μL) | 814.00 (500.63–935.00) | 229.00 (146.50–295.10) | 49.00 (33.00–100.38) | <0.001 |
| Natural killer T cell count (/μL) | 49.00 (33.00–100.38) | 83.50 (62.25–135.50) | 38.00 (15.00–66.00) | 0.017 |
| B lymphocyte count (/μL) | 38.00 (15.00–66.00) | 189.00 (131.00–285.94) | 130.00 (45.69–266.00) | 0.256 |
| Th/Tc (Helper T cell/ Cytotoxic T cell ) | 130.00 (45.69–266.00) | 1.92 (1.22–2.98) | 1.92 (1.34–2.92) | 0.895 |
| IgG (g/L) | 48.00 (36.25-58.00) | 12.10 (10.43-13.35) | 11.65 (10.10-14.68) | 0.887 |
| IgA (g/L) | 11.65 (10.10-14.68) | 2.51 (1.86-2.91) | 2.22 (1.62-2.73) | 0.514 |
| IgM (g/L) | 2.22 (1.62-2.73) | 0.84 (0.68-1.14) | 0.89 (0.73-1.03) | 0.879 |
| IgE (g/L) | 0.89 (0.73-1.03) | 47.20 (10.21-108.50) | 70.20 (34.20-185.00) | 0.281 |
| C3 (g/L) | 70.20 (34.20-185.00) | 0.99 (0.84-1.08) | 0.94 (0.70-1.02) | 0.281 |
| C4 (g/L) | 0.94 (0.70-1.02) | 0.23 (0.18-0.29) | 0.22 (0.17-0.30) | 0.638 |
| Interleukin 1β (pg/mL) | 0.22 (0.17–0.30) | 4.06 (1.94–10.25) | 2.72 (1.95–7.84) | 0.419 |
| Interleukin 2 (pg/mL) | 2.72 (1.95–7.84) | 2.60 (2.10–4.21) | 2.86 (2.05–4.31) | 0.735 |
| Interleukin 4 (pg/mL) | 2.86 (2.05–4.31) | 2.27 (2.10–2.72) | 2.33 (1.93–2.48) | 0.855 |
| Interleukin 5 (pg/mL) | 2.33 (1.93–2.48) | 1.83 (1.48–2.95) | 2.38 (1.74–6.13) | 0.241 |
| Interleukin 6 (pg/mL) | 2.38 (1.74–6.13) | 12.84 (7.81–26.46) | 120.66 (28.90–216.00) | <0.001 |
| Interleukin 8 (pg/mL) | 120.66 (28.90–216.00) | 11.69 (5.18–58.03) | 33.56 (15.18–90.93) | 0.201 |
| Interleukin 9 (pg/mL) | 33.56 (15.18–90.93) | 1.29 (0.90–1.49) | 1.13 (1.08–2.40) | 0.893 |
| Interleukin 10 (pg/mL) | 1.13 (1.08–2.40) | 3.29 (2.32–4.70) | 3.16 (2.59–7.35) | 0.787 |
| Interleukin 12P70 (pg/mL) | 3.16 (2.59–7.35) | 2.17 (1.91–2.85) | 2.02 (1.80–2.61) | 0.285 |
| Interleukin 13 (pg/mL) | 2.02 (1.80–2.61) | 1.71 (0.46–3.83) | 0.64 (0.52–0.83) | 0.671 |
| Interleukin 17 (pg/mL) | 0.64 (0.52–0.83) | 4.91 (2.56–7.15) | 6.22 (3.01–8.87) | 0.449 |
| Interferon α (pg/mL) | 6.22 (3.01–8.87) | 2.64 (1.84–5.58) | 2.72 (2.14–4.30) | 0.804 |
| Interferon γ (pg/mL) | 2.72 (2.14–4.30) | 4.42 (2.39–9.15) | 4.50 (2.60–17.38) | 0.489 |
| Tumor necrosis factor α (pg/mL) | 4.50 (2.60–17.38) | 2.27 (1.59–4.24) | 2.44 (1.49–4.87) | 0.888 |
| Granulocyte colony stimulating factor (pg/mL) | 2.44 (1.49–4.87) | 1.03 (0.92–1.31) | 1.32 (1.06–1.92) | 0.045 |
| Granulocyte macrophage colony stimulating factor (pg/mL) | 1.32 (1.06–1.92) | 2.01 (1.91–2.04) | 1.86 (1.61–3.08) | 0.977 |
| Vascular endothelial growth factor (pg/mL) | 1.86 (1.61–3.08) | 125.43 (5.07–234.31) | 205.47 (71.34–257.00) | 0.323 |
| Macrophage inflammatory protein 1α (pg/mL) | 205.47 (71.34–257.00) | 14.70 (6.97–28.47) | 11.92 (6.14–24.15) | 0.894 |
| Monocyte chemoattractant protein 1 (pg/mL) | 11.92 (6.14–24.15) | 49.20 (4.26–96.73) | 110.45 (6.27–227.81) | 0.097 |
